# Supplementary material for: Roosters do not warn the bird in the mirror: The cognitive ecology of mirror self-recognition
Source: PLoS One. 2023 Oct 25;18(10):e0291416. doi: 10.1371/journal.pone.0291416 (PMC10599514; doi:10.1371/journal.pone.0291416)
Supplement: S1 Table — Results of the comparison of behaviors during habituation sessions with a mirror and sessions without a mirror. Given are Median (Mdn), interquartile-range (IQR), mean (M), standard deviation (SD) and test results of non-parametric Mann-Whitney-U-test. (DOCX) [file pone.0291416.s001.docx]

**S1 Table. Habituation analysis between mirror- and no-mirror sessions.** Results of the comparison of behaviors during habituation sessions with a mirror and sessions without a mirror. Given are Median (Mdn), interquartile-range (IQR), mean (M), standard deviation (SD) and test results of non-parametric Mann-Whitney-U-test.

|  | **Mirror** | | | | **No-mirror** | | | |  |
| --- | --- | --- | --- | --- | --- | --- | --- | --- | --- |
| **Behavior** | **Mdn** | **IQR** | **M** | **SD** | **Mdn** | **IQR** | **M** | **SD** | **Statistics** |
| Time mirror (s) | 557.11 | 460.62 – 652.67 | 551.53 | 151.18 | 525.00 | 420.37 – 602.53 | 511.92 | 121.89 | n = 99, U = 1354.00, p = 0.110 |
| Fights mirror/grid | 0.00 | 0.00 – 0.00 | 0.05 | 0.25 | 0.00 | 0.00 – 0.00 | 0.00 | 0.00 | n = 99, U = 1189.00, p = 0.196 |
| Pecks mirror/grid | 0.00 | 0.00 – 0.00 | 0.41 | 1.45 | 0.00 | 0.00 – 2.75 | 1.28 | 2.09 | n = 99, U = 855.50, p = 0.003 |
| Pecks floor | 11.00 | 4.00 – 22.00 | 17.03 | 21.00 | 14.50 | 8.25 – 27.50 | 21.14 | 19.94 | n = 99, U = 940.00, p = 0.158 |
| Pecks wall | 1.00 | 0.00 – 6.00 | 3.06 | 4.02 | 0.00 | 0.00 – 1.00 | 0.61 | 1.08 | n = 99, U = 1581.00, p < 0.001 |
| Crowing | 3.00 | 1.00 – 11.00 | 6.30 | 6.86 | 2.50 | 0.00 – 9.00 | 5.06 | 5.51 | n = 99, U = 1256.50, p = 0.367 |
| Plumage ruffling | 2.00 | 1.00 – 3.00 | 2.06 | 1.82 | 1.00 | 1.00 – 3.00 | 1.67 | 1.47 | n = 99, U = 1260.00, p = 0.349 |
| Head shaking | 4.00 | 2.00 – 6.00 | 4.79 | 4.54 | 3.00 | 2.00 – 4.00 | 3.72 | 3.11 | n = 99, U = 1313.50, p = 0.187 |
| Head turning | 25.00 | 15.00 – 45.00 | 30.13 | 20.55 | 16.00 | 11.25 – 24.00 | 18.56 | 10.68 | n = 99, U = 1546.00, p = 0.003 |
| Preening mark_mirror | 0.00 | 0.00 – 1.00 | 0.70 | 1.30 | 0.00 | 0.00 – 0.00 | 0.42 | 1.36 | n = 99, U = 1267.00, p = 0.221 |
| Preening mark_away | 0.00 | 0.00 – 0.00 | 0.44 | 1.06 | 0.00 | 0.00 – 0.75 | 0.69 | 1.47 | n = 99, U = 1061.00, p = 0.457 |
| Preening other_mirror | 2.00 | 0.00 – 8.00 | 4.17 | 4.75 | 1.00 | 0.00 – 4.00 | 3.00 | 4.82 | n = 99, U = 1312.00, p = 0.185 |
| Preening other_away | 2.00 | 0.00 – 9.0 | 4.89 | 5.88 | 1.00 | 0.00 – 5.00 | 3.81 | 5.44 | n = 99, U = 1271.50, p = 0.310 |
| Turning clockwise | 1.00 | 0.00 – 3.00 | 2.57 | 3.71 | 0.50 | 0.00 – 2.00 | 1.78 | 2.89 | n = 99, U = 1267.00, p = 0.308 |
| Turning anticlockwise | 3.00 | 0.00 – 6.00 | 3.78 | 4.23 | 2.00 | 0.00 – 5.00 | 3.92 | 6.08 | n = 99, U = 1180.00, p = 0.732 |
