# Supplementary material for: Roosters do not warn the bird in the mirror: The cognitive ecology of mirror self-recognition
Source: PLoS One. 2023 Oct 25;18(10):e0291416. doi: 10.1371/journal.pone.0291416 (PMC10599514; doi:10.1371/journal.pone.0291416)
Supplement: S2 Table — Results of the comparison of the different mirror exposure sessions 1–4 during habituation. Given are Median (Mdn), interquartile-range (IQR), mean (M), standard deviation (SD) and test results of non-parametric Friedmann-Test, followed by the Dunn-Bonferroni Post-hoc test giving z- and p-values. (DOCX) [file pone.0291416.s002.docx]

**S2 Table. Habituation analysis between multiple mirror sessions.** Results of the comparison of the different mirror exposure sessions 1-4 during habituation. Given are Median (Mdn), interquartile-range (IQR), mean (M), standard deviation (SD) and test results of non-parametric Friedmann-Test, followed by the Dunn-Bonferroni Post-hoc test giving *z-* and *p*-values.

|  | **Mirror 1** | | **Mirror 2** | | | | **Mirror 3** | | | **Mirror 4** | | |  |
| --- | --- | --- | --- | --- | --- | --- | --- | --- | --- | --- | --- | --- | --- |
| **Behavior** | **Mdn**  **IQR** | **M SD** | | **Mdn**  **IQR** | **M SD** | **Mdn**  **IQR** | | **M SD** | **Mdn**  **IQR** | | **M SD** | **Statistics** | |
| Time mirror (s) | 543.73  436.56 – 690.70 | 559.12 166.09 | | 565.24  521.13 – 673.96 | 574.87 152.73 | 564.61  455.52 – 628.26 | | 552.82 126.95 | 519.71  431.52 – 596.53 | | 509.26 171.75 | n = 9, χ²(3) = 2.333, p = 0.506 | |
| Fights mirror | 0.00  0.00 – 0.00 | 0.00 0.00 | | 0.00  0.00 – 0.00 | 0.17 0.38 | 0.00  0.00 – 0.00 | | 0.00 0.00 | 0.00  0.00 – 0.00 | | 0.00 0.00 | n = 9, χ²(3) = 6.000, p = 0.112 | |
| Pecks mirror | 0.00  0.00 – 0.00 | 0.50 1.40 | | 0.00  0.00 – 0.00 | 0.67 2.38 | 0.00  0.00 – 0.00 | | 0.22 0.55 | 0.00  0.00 – 0.50 | | 0.23 0.44 | n = 9, χ²(3) = 1.457, p = 0.692 | |
| Pecks floor | 4.50  0.00 – 20.50 | 9.93 11.68 | | 11.00  4.00 – 20.25 | 17.28 19.21 | 17.00  8.75 – 23.25 | | 25.67 29.97 | 6.00  3.00 – 23.50 | | 12.38 11.85 | n = 9, χ²(3) = 8.932, p = 0.030 | |
| Pecks wall | 2.00  0.00 – 6.00 | 4.14 5.16 | | 1.50  0.00 – 5.25 | 2.50 2.89 | 1.00  0.00 – 5.25 | | 3.17 4.66 | 1.00  0.00 – 6.50 | | 2.54 3.18 | n = 9, χ²(3) = 0.591, p = 0.899 | |
| Crowing | 3.00  0.00 – 14.00 | 7.00 7.72 | | 2.00  1.00 – 10.75 | 5.89 6.78 | 6.50  0.00 – 12.00 | | 7.39 7.90 | 2.00  1.00 – 9.50 | | 4.61 4.39 | n = 9, χ²(3) = 2.639, p = 0.451 | |
| Plumage ruffling | 1.50  1.00 – 2.00 | 1.93 1.86 | | 2.50  1.00 – 4.25 | 2.72 2.11 | 2.00  0.75 – 3.25 | | 2.17 1.76 | 1.00  0.00 – 2.00 | | 1.15 1.07 | n = 9, χ²(3) = 2.507, p = 0.474 | |
| Head shaking | 4.00  0.75 – 5.50 | 5.00 6.54 | | 4.00  1.00 – 6.00 | 3.67 2.50 | 4.50  2.75 – 7.00 | | 5.67 4.89 | 4.00  3.00 – 5.50 | | 4.92 3.80 | n = 9, χ²(3) = 3.453, p = 0.327 | |
| Head turn | 41.00  19.75 – 50.75 | 37.86 20.16 | | 27.00  20.50 – 45.50 | 36.06 24.95 | 25.00  15.75 – 47.00 | | 29.94 15.92 | 15.00  5.00 – 20.00 | | 13.85 9.49 | n = 9, χ²(3) = 8.420, p = 0.038 | |
| Preening mark_mirror | 0.00  0.00 – 1.00 | 0.36 0.63 | | 0.00  0.00 – 3.00 | 1.17 1.76 | 0.00  0.00 – 0.00 | | 0.39 0.98 | 0.00  0.00 – 1.00 | | 0.85 1.40 | n = 9, χ²(3) = 8.786, p = 0.032 | |
| Preening mark_away | 0.00  0.00 – 0.00 | 0.36 0.93 | | 0.00  0.00 – 0.50 | 0.56 1.15 | 0.00  0.00 – 0.00 | | 0.44 1.25 | 0.00  0.00 – 0.50 | | 0.38 0.87 | n = 9, χ²(3) = 1.182, p = 0.757 | |
| Preening other_mirror | 2.50  0.00 – 6.25 | 3.79 4.81 | | 3.00  0.00 – 11.25 | 5.00 5.36 | 1.00  0.00 – 4.75 | | 3.17 4.42 | 6.00  0.50 – 8.00 | | 4.85 4.47 | n = 9, χ²(3) = 2.788, p = 0.425 | |
| Preening other_away | 1.50  0.00 – 4.50 | 2.86 3.53 | | 3.50  1.00 – 9.25 | 6.22 6.37 | 2.00  0.75 – 13.75 | | 6.44 7.51 | 3.00  0.00 – 5.00 | | 3.08 3.62 | n = 9, χ²(3) = 6.303, p = 0.098 | |
| Turning clockwise | 1.00  0.00 – 5.25 | 2.79 3.70 | | 1.00  0.00 – 6.25 | 3.22 4.78 | 0.50  0.00 – 3.00 | | 1.67 2.09 | 1.00  0.00 – 4.00 | | 2.69 3.94 | n = 9, χ²(3) = 4.522, p = 0.210 | |
| Turning anticlockwise | 2.00  0.75 – 3.25 | 2.14 1.87 | | 1.00  0.00 – 7.50 | 4.06 5.41 | 4.00  0.75 – 6.00 | | 4.28 3.85 | 3.00  1.00 – 7.50 | | 4.46 4.72 | n = 9, χ²(3) = 5.812, p = 0.121 | |

| **Behavior** | **Sample** | ***z-*value** | **p-value** |
| --- | --- | --- | --- |
| Pecks floor | Mirror1 vs. Mirror2 | -0.889 | 0.865 |
|  | Mirror1 vs. Mirror3 | -1.778 | 0.021 |
|  | Mirror1 vs. Mirror4 | -0.667 | 1.000 |
|  | Mirror2 vs. Mirror3 | -0.889 | 0.865 |
|  | Mirror2 vs. Mirror4 | -0.222 | 1.000 |
|  | Mirror3 vs. Mirror4 | -1.111 | 0.407 |
| Head turn | Mirror1 vs. Mirror2 | -0.278 | 1.000 |
|  | Mirror1 vs. Mirror3 | -0.167 | 1.000 |
|  | Mirror1 vs. Mirror4 | -1.556 | 0.064 |
|  | Mirror2 vs. Mirror3 | -0.111 | 1,000 |
|  | Mirror2 vs. Mirror4 | -1.278 | 0.215 |
|  | Mirror3 vs. Mirror4 | -1.389 | 0.135 |
| Preening mark_mirror | Mirror1 vs. Mirror2 | -1.000 | 0.602 |
|  | Mirror1 vs. Mirror3 | -0.111 | 1.000 |
|  | Mirror1 vs. Mirror4 | -1.111 | 0.407 |
|  | Mirror2 vs. Mirror3 | -0.889 | 0.865 |
|  | Mirror2 vs. Mirror4 | -0.111 | 1.000 |
|  | Mirror3 vs. Mirror4 | -1.000 | 0.602 |
