# Supplementary material for: Roosters do not warn the bird in the mirror: The cognitive ecology of mirror self-recognition
Source: PLoS One. 2023 Oct 25;18(10):e0291416. doi: 10.1371/journal.pone.0291416 (PMC10599514; doi:10.1371/journal.pone.0291416)
Supplement: S3 Table — (DOCX) [file pone.0291416.s003.docx]

| **Subject** | **touch-region** | **1** | **2** | **3** | **4** | **χ², *p*, Cramér’s V** |
| --- | --- | --- | --- | --- | --- | --- |
| **B1** | Mark | 2_a_ | 0_a_ | 3_a_ | 0_a_ | χ² = 2.441, *p* = 0.530, Cramér’s V = 0.272 |
|  | Other | 5_a_ | 5_a_ | 15_a_ | 3_a_ |  |
| **B2** | Mark | 3_a_ | 3_a_ | 3_a_ | 4_a_ | χ² = 0.530, *p* = 0.926, Cramér’s V = 0.091 |
|  | Other | 11_a_ | 17_a_ | 10_a_ | 13_a_ |  |
| **B3** | Mark | 7_a_ | 13_a_ | 5_a_ | 6_a_ | χ² = 0.650, *p* = 0.889, Cramér’s V = 0.084 |
|  | Other | 14_a_ | 22_a_ | 14_a_ | 12_a_ |  |
| **B4** | Mark | 8_a_ | 5_a_ | 3_a_ | 2_a_ | χ² = 0.109, *p* = 1.000, Cramér’s V = 0.040 |
|  | Other | 20_a_ | 15_a_ | 9_a_ | 5_a_ |  |
| **B5** | Mark | 0_a_ | 2_a_ | 8_a_ | 0_a_ | χ² = 2.941, *p* = 0.411, Cramér’s V = 0.308 |
|  | Other | 2_a_ | 6_a_ | 11_a_ | 2_a_ |  |
| **LB1** | Mark | 0 | 0 | 0 | 0 | N/A |
|  | Other | 0 | 5_a_ | 2_a_ | 7_a_ |  |
| **LB2** | Mark | 0_a_ | 0 | 3_a_ | 0 | χ² = 8.743, *p* = 0.029, Cramér’s V = 0.717 |
|  | Other | 5_a_ | 0 | 2_a_ | 7_a_ |  |
| **LB3** | Mark | 1_a_ | 9_a_ | 5_a_ | 1_a_ | χ² = 3.997, *p* = 0.299, Cramér’s V = 0.289 |
|  | Other | 5_a_ | 10_a_ | 10_a_ | 7_a_ |  |
| **LB4** | Mark | 0 | 1_a_ | 1_a_ | 0 | χ² = 0.058, *p* = 1.000, Cramér’s V = 0.091 |
|  | Other | 0 | 2_a_ | 3_a_ | 0 |  |
| **LB5** | Mark | 5_a_ | 2_a_ | 1_a_ | 1_a_ | χ² = 3.098, *p* = 0.458, Cramér’s V = 0.254 |
|  | Other | 10_a_ | 14_a_ | 9_a_ | 6_a_ |  |
| **LB6** | Mark | 4_a_ | 1_a_ | 2_a_ | 0_a_ | χ² = 2.748, *p* = 0.517, Cramér’s V = 0.303 |
|  | Other | 8_a_ | 7_a_ | 4_a_ | 4_a_ |  |
| **LB7** | Mark | 10_a_ | 4_a_ | 2_a_ | 2_a_ | χ² = 2.748, *p* = 0.444, Cramér’s V = 0.204 |
|  | Other | 16_a_ | 16_a_ | 9_a_ | 7_a_ |  |
| **LB8** | Mark | 0 | 1_a_ | 0_a_ | 2_a_ | χ² = 3.333, *p* = 0.246, Cramér’s V = 0.471 |
|  | Other | 0 | 2_a_ | 7_a_ | 3_a_ |  |
| **LB9** | Mark | 3_a_ | 0_a_ | 1_a_ | 1_a_ | χ² = 1.163, *p* = 0.903, Cramér’s V = 0.235 |
|  | Other | 8_a_ | 3_a_ | 3_a_ | 2_a_ |  |
| **M1** | Mark | 4_a_ | 3_a_ | 0_a_ | 4_a_ | χ² = 1.244, *p* = 0.832, Cramér’s V = 0.179 |
|  | Other | 7_a_ | 7_a_ | 2_a_ | 12_a_ |  |
| **M2** | Mark | 0_a_ | 0_a_ | 2_a_ | 1_a_ | χ² = 3.052, *p* = 0.375, Cramér’s V = 0.233 |
|  | Other | 5_a_ | 22_a_ | 19_a_ | 7_a_ |  |
| **M3** | Mark | 3_a_ | 0_a_ | 0_a_ | 0_a_ | χ² = 3.850, *p* = 0.357, Cramér’s V = 0.428 |
|  | Other | 7_a_ | 2_a_ | 6_a_ | 3_a_ |  |
| **M4** | Mark | 7_a_ | 3_a_ | 5_a_ | 11_a_ | χ² = 1.526, *p* = 0.716, Cramér’s V = 0.126 |
|  | Other | 18_a_ | 6_a_ | 22_a_ | 24_a_ |  |

**S3 Table. Analyses at individual level for touches of the mark region and touches of other parts of the body during the mark test.**

Results of the Chi-square-test (χ², p-values, Cramér’s V) at the individual level for touches of the mark region and touches of other body regions in conditions 1 (mirror + mark), 2 (mirror + sham mark), 3 (no mirror + mark), 4 (no mirror + sham mark) during the mark test. N/A: not applicable.
